# Supplementary material for: Large, regionally variable shifts in diatom and dinoflagellate biomass in the North Atlantic over six decades
Source: PLoS One. 2025 Jun 4;20(6):e0323675. doi: 10.1371/journal.pone.0323675 (PMC12136357; doi:10.1371/journal.pone.0323675)
Supplement: S1 Text — (DOCX) [file pone.0323675.s001.docx]

**Supporting Methods**

*Model assessment using posterior predictive checks*

The standard approach to Bayesian model validation is posterior predictive model checking (Gelman et al. 1996; 2013), which draws on the ability to approximate the posterior predictive distribution, i.e., the distribution of unobserved values conditional on observed data. The posterior predictive distribution of an observable $\tilde{y}$ conditional on observed data $y$ is defined by

| $p\left( \tilde{y}\vert y \right)=\int L\left( \tilde{y}\vert\boldsymbol{\theta} \right)p\left( \boldsymbol{\theta}\vert y \right)d\boldsymbol{\theta}$ | (S1) |
| --- | --- |

where $L\left( y|\boldsymbol{\theta} \right)$ and $p\left( \boldsymbol{\theta} \right)$ are respectively the likelihood and the prior in the Bayesian model. The posterior predictive distribution is esssentially the likelihood of $\tilde{y}$ integrated over the posterior uncertainty in $\boldsymbol{\theta}$. The optimal Bayesian prediction under a quadratic loss function is the posterior predictive mean value $E[\tilde{y}|y]$.

The rationale of posterior predictive model checking is to compare the observed data with replicated data simulated from the posterior predictive distribution, or alternatively to compare some test quantity $T(y,\boldsymbol{\theta})$ based on the observed data, to the same statistic $T(y^{rep},\boldsymbol{\theta})$ for replicated data from the posterior predictive distribution. If the model fits the data well, then replicated data generated under the model should look similar to the observed data. Therefore, systematic discrepancies between $T(y,\boldsymbol{\theta})$ and $\left( y^{rep},\boldsymbol{\theta} \right)$ indicate model misfit. The comparison of $T(y,\boldsymbol{\theta})$ to $\left( y^{rep},\boldsymbol{\theta} \right)$ may be visual using graphical tools such as histograms, or formal using posterior predictive $p$-values also known as Bayesian $p$-values defined as (Gelman et al. 1996; 20013)

$P_{B}=Pr\left( T(y^{rep},\theta)\geq T\left( y,\theta\right|y \right)$ (S2)

If observed data are consistent with model predictions, then *P_B_* should be close to 0.50. Values of *P_B_* close to 0 or 1 provide evidence for model inadequacy with *P_B_* close to 0 indicating a lack of fit and values close to 1 pointing to overfitting, which arises when a model is unnecessarily too complex. In the Bayesian MCMC framework, a simulation-based approximation of $P_{B}$ is given by the proportion of posterior predictive data replicates for which the test quantity exceeds its original data counterpart. At the most basic level, posterior predictive checks involve the comparison of observed data to their posterior predictions. Relevant test quantities can differ depending on the assumed sampling distribution. For the normal likelihood assumed here to describe the logit of the proportion of total (diatom + dinoflagellate) biomass due to diatoms and the log biomass of each functional type, the relevant test statistics are the mean and the standard deviation, which fully characterize the normal distribution.

**Supporting Results**

*Counts of non-zero observations of diatom and dinoflagellate biomass in the CPR data at different spatial resolutions*

The raw CPR data analysed here had 304,472 observations of diatoms and 217,232 observations of dinoflagellates, but most of these observations did not have simultaneous counts for both diatoms and dinoflagellates. When aggregated to 1° x 1° monthly resolution, the number of diatom and dinoflagellate biomass observations per month ranged from 2,400 to 40,000 (S1 Table). In about half the year, the number of diatom biomass observations was double (or more) the number of dinoflagellate biomass observations, resulting in poor estimates of the biomass ratio at this resolution. When the aggregation was increased to 2.5° latitude, five provinces, and monthly the total number of observations per province and month dropped considerably (S2 Table) but the number of diatom and dinoflagellate observations were much more balanced. The number of observations of diatom and dinoflagellate biomass at this aggregation level varied within years and across decades, with the smallest number of observations arising in the winter months. A notable feature was the near-total absence of data for the 1980s in the NWCS (S10 Fig.).
