# Supplementary material for: Large, regionally variable shifts in diatom and dinoflagellate biomass in the North Atlantic over six decades
Source: PLoS One. 2025 Jun 4;20(6):e0323675. doi: 10.1371/journal.pone.0323675 (PMC12136357; doi:10.1371/journal.pone.0323675)
Supplement: S1 Table — The number of observations of diatom and dinoflagellate biomass for the CPR data aggregated by month at 1° x 1° spatial resolution. (DOCX) [file pone.0323675.s003.docx]

**Table S1.** The number of observations of diatom and dinoflagellate biomass for the CPR data aggregated by month at 1° x 1° spatial resolution.

|  | **JAN** | **FEB** | **MAR** | **APR** | **MAY** | **JUNE** | **JUL** | **AUG** | **SEPT** | **OCT** | **NOV** | **DEC** |
| --- | --- | --- | --- | --- | --- | --- | --- | --- | --- | --- | --- | --- |
| **DIATOM** | 8167 | 11952 | 28418 | 46906 | 48554 | 34292 | 24138 | 19989 | 24283 | 27376 | 19119 | 11278 |
| **DINOFL.** | 3208 | 2446 | 4305 | 9776 | 20971 | 30857 | 39379 | 36999 | 30621 | 22762 | 10681 | 5227 |
