# Supplementary material for: Large, regionally variable shifts in diatom and dinoflagellate biomass in the North Atlantic over six decades
Source: PLoS One. 2025 Jun 4;20(6):e0323675. doi: 10.1371/journal.pone.0323675 (PMC12136357; doi:10.1371/journal.pone.0323675)
Supplement: S2 Table — The number of observations of diatom (upper number) and dinoflagellate (lower number) biomass for the CPR data aggregated by month over 2.5° latitude bands for each biogeographic province. (DOCX) [file pone.0323675.s004.docx]

**Table S2.** The number of observations of diatom (upper number) and dinoflagellate (lower number) biomass for the CPR data aggregated by month over 2.5° latitude bands for each biogeographic province.

|  | **JAN** | **FEB** | **MAR** | **APR** | **MAY** | **JUNE** | **JUL** | **AUG** | **SEPT** | **OCT** | **NOV** | **DEC** |
| --- | --- | --- | --- | --- | --- | --- | --- | --- | --- | --- | --- | --- |
| **ARCT** | 94  30 | 65  24 | 103  15 | 162  24 | 229  88 | 251  174 | 253  227 | 242  216 | 254  189 | 220  160 | 208  121 | 162  69 |
| **SARC** | 36  14 | 62  15 | 128  46 | 168  93 | 191  144 | 185  178 | 172  180 | 163  173 | 173  180 | 168  160 | 156  130 | 97  52 |
| **NWCS** | 139  130 | 133  111 | 146  111 | 167  123 | 165  133 | 156  139 | 132  143 | 111  147 | 133  157 | 147  149 | 151  137 | 154  140 |
| **NADR** | 115  39 | 164  51 | 230  105 | 299  194 | 287  240 | 273  274 | 256  264 | 236  263 | 250  266 | 240  236 | 218  188 | 172  92 |
| **NECS** | 221  114 | 268  127 | 330  180 | 347  288 | 349  334 | 338  335 | 344  345 | 337  343 | 326  325 | 317  299 | 305  235 | 264  171 |
