# Supplementary material for: Large, regionally variable shifts in diatom and dinoflagellate biomass in the North Atlantic over six decades
Source: PLoS One. 2025 Jun 4;20(6):e0323675. doi: 10.1371/journal.pone.0323675 (PMC12136357; doi:10.1371/journal.pone.0323675)
Supplement: S3 Table — Number of biomass ratios, diatom biomasses (observed/missing), dinoflagellate biomasses (observed/missing), the latitude range of observation locations, and the number of missing temperature data in spatially aggregated data, grouped by biogeographic province. (DOCX) [file pone.0323675.s005.docx]

**Table S3.** Summary of missing observations after spatial aggregation into 2.5° latitude bins. Number of biomass ratios, diatom biomasses (observed/missing), dinoflagellate biomasses (observed/missing), the latitude range of observation locations, and the number of missing temperature data in spatially aggregated data, grouped by biogeographic province.

| **Province** | **Biomass ratio (n)** | **Diatoms (n / missing)** | **Dinoflagellates (n / missing)** | **Latitude range** | **SST (# missing)** |
| --- | --- | --- | --- | --- | --- |
| ARCT | 2324 | 2243 / 81 | 1337 / 987 | (50, 80) | 0 |
| SARC | 1784 | 1699 / 85 | 1365 / 419 | (57.5, 72.5) | 0 |
| NWCS | 1859 | 1734 / 125 | 1620 / 239 | (30, 52.5) | 2 |
| NADR | 2947 | 2740 / 207 | 2212 / 735 | (42.5, 57.5) | 0 |
| NECS | 3837 | 3746 / 91 | 3096 / 741 | (45, 65) | 0 |
