# Supplementary material for: Large, regionally variable shifts in diatom and dinoflagellate biomass in the North Atlantic over six decades
Source: PLoS One. 2025 Jun 4;20(6):e0323675. doi: 10.1371/journal.pone.0323675 (PMC12136357; doi:10.1371/journal.pone.0323675)
Supplement: S4 Table — Average annual rate of change in the diatom index (diatom/(diatom + dinoflagellate) biomass ratio, logit scale) and in the biomass of each functional groups and the total of both groups (natural log scale) across the five biogeographic provinces estimated from the Time-Space-Temperature models, expressed as a % change per year ± half the width of the 95% credible interval. An effect (slope) of 0.01 a–1 corresponds to approximately a 1% change in biomass or in the biomass ratio per year. (DOCX) [file pone.0323675.s006.docx]

**Table S4.** Average annual rate of change in the diatom index (diatom/(diatom + dinoflagellate) biomass ratio, logit scale) and in the biomass of each functional type and the total of both groups (natural log scale) across the five biogeographic provinces estimated from the Time-Space-Temperature models, expressed as a % change per year ± half the width of the 95% credible interval. An effect (slope) of 0.01 a^–1^ corresponds to approximately a 1% change in biomass or in the biomass ratio per year.

|  | **ARCT** | **SARC** | **NWCS** | **NADR** | **NECS** |
| --- | --- | --- | --- | --- | --- |
| Diatom index | -0.935 ± 0.51 | 1.1 ± 0.69 | 0.76 ± 0.79 | 0.75 ± 0.55 | 2.5 ± 0.44 |
| Diatom biomass | -0.7 ± 0.53 | -0.93 ± 0.57 | 2.2 ± 0.6 | -0.245 ± 0.36 | 1.8 ± 0.49 |
| Dinoflagellate biomass | 0.245 ± 0.4 | -2.2 ± 0.41 | 1.2 ± 0.58 | -1.1 ± 0.33 | -0.79 ± 0.31 |
| Total biomass | -0.16 ± 0.4 | -1.4 ± 0.36 | 1.1 ± 0.49 | -0.59 ± 0.33 | 0.575 ± 0.33 |
