# Supplementary material for: Large, regionally variable shifts in diatom and dinoflagellate biomass in the North Atlantic over six decades
Source: PLoS One. 2025 Jun 4;20(6):e0323675. doi: 10.1371/journal.pone.0323675 (PMC12136357; doi:10.1371/journal.pone.0323675)
Supplement: S5 Table — Posterior median of the annual mean diatom index (dimensionless) and phytoplankton biomass (diatom, dinoflagellate, total) (g C) ± half the width of the 95% credible interval for each province. (DOCX) [file pone.0323675.s007.docx]

**Table S5.** Posterior median of the annual mean diatom index (dimensionless) and phytoplankton biomass (diatom, dinoflagellate, total) (g C) ± half the width of the 95% credible interval.

|  | **ARCT** | **SARC** | **NWCS** | **NADR** | **NECS** |
| --- | --- | --- | --- | --- | --- |
| Diatom index | 1.2 ± 0.17 | -0.93 ± 0.3 | -0.93 ± 0.3 | -0.77 ± 0.19 | -0.28 ± 0.15 |
| Diatom biomass | 4.7 ± 0.16 | 5 ± 0.21 | 4.9 ± 0.24 | 4.6 ± 0.13 | 5.9 ± 0.17 |
| Dinoflagellate biomass | 3.5 ± 0.16 | 5.7 ± 0.14 | 5.9 ± 0.22 | 5.4 ± 0.11 | 6.2 ± 0.1 |
| Total biomass | 5.5 ± 0.16 | 6.8 ± 0.14 | 7.2 ± 0.16 | 6.4 ± 0.11 | 7.5 ± 0.11 |
