# Supplementary material for: Large, regionally variable shifts in diatom and dinoflagellate biomass in the North Atlantic over six decades
Source: PLoS One. 2025 Jun 4;20(6):e0323675. doi: 10.1371/journal.pone.0323675 (PMC12136357; doi:10.1371/journal.pone.0323675)
Supplement: S6 Table — Proportion of variation in the data explained by Time-Space-Temperature models (Bayesian R2) from the full model and after excluding each explanatory variable one at a time. (DOCX) [file pone.0323675.s008.docx]

**Table S6.** Proportion of variation in the data explained by Time-Space-Temperature models (Bayesian *R*^2^) from the full model and after excluding each explanatory variable one at a time.

| Excluded variable | Model | | |
| --- | --- | --- | --- |
|  | Diatom index  (Logit) | Log biomass | |
|  |  | Diatom | Dinoflagellate |
| None (Full model) | 0.37 | 0.32 | 0.64 |
| SST | 0.34 | 0.22 | 0.41 |
| Latitude | 0.36 | 0.30 | 0.62 |
| Month | 0.06 | - | - |
