# Supplementary figures and images for: Large, regionally variable shifts in diatom and dinoflagellate biomass in the North Atlantic over six decades
Source: PLoS One. 2025 Jun 4;20(6):e0323675. doi: 10.1371/journal.pone.0323675 (PMC12136357; doi:10.1371/journal.pone.0323675)

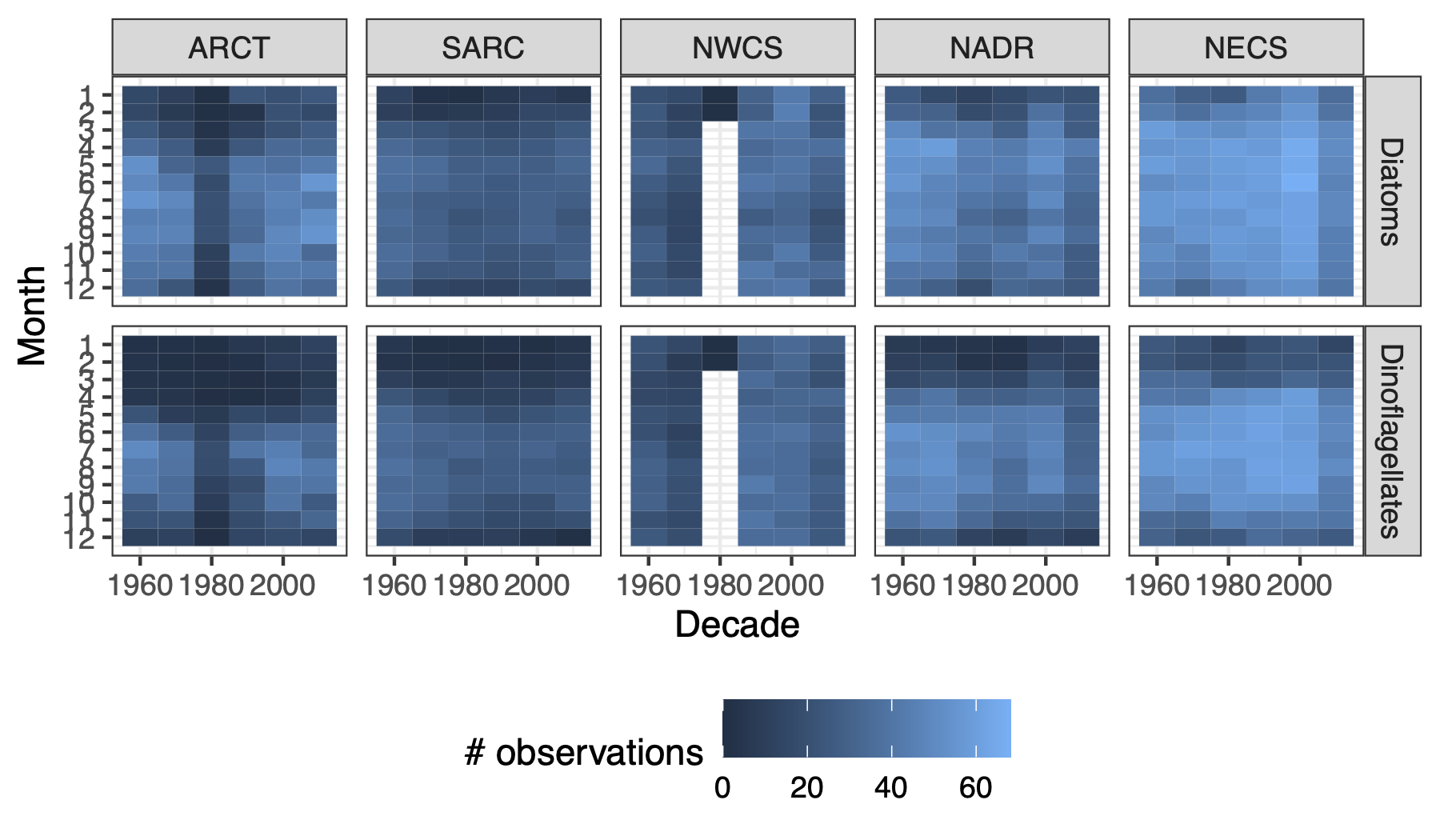

Supplement: S1 Fig — Number of observations of diatom (top panels) or dinoflagellate (bottom panels) biomass after aggregation into 2.5° latitude bins in each province by month and decade. (PNG) [file pone.0323675.s009.png]

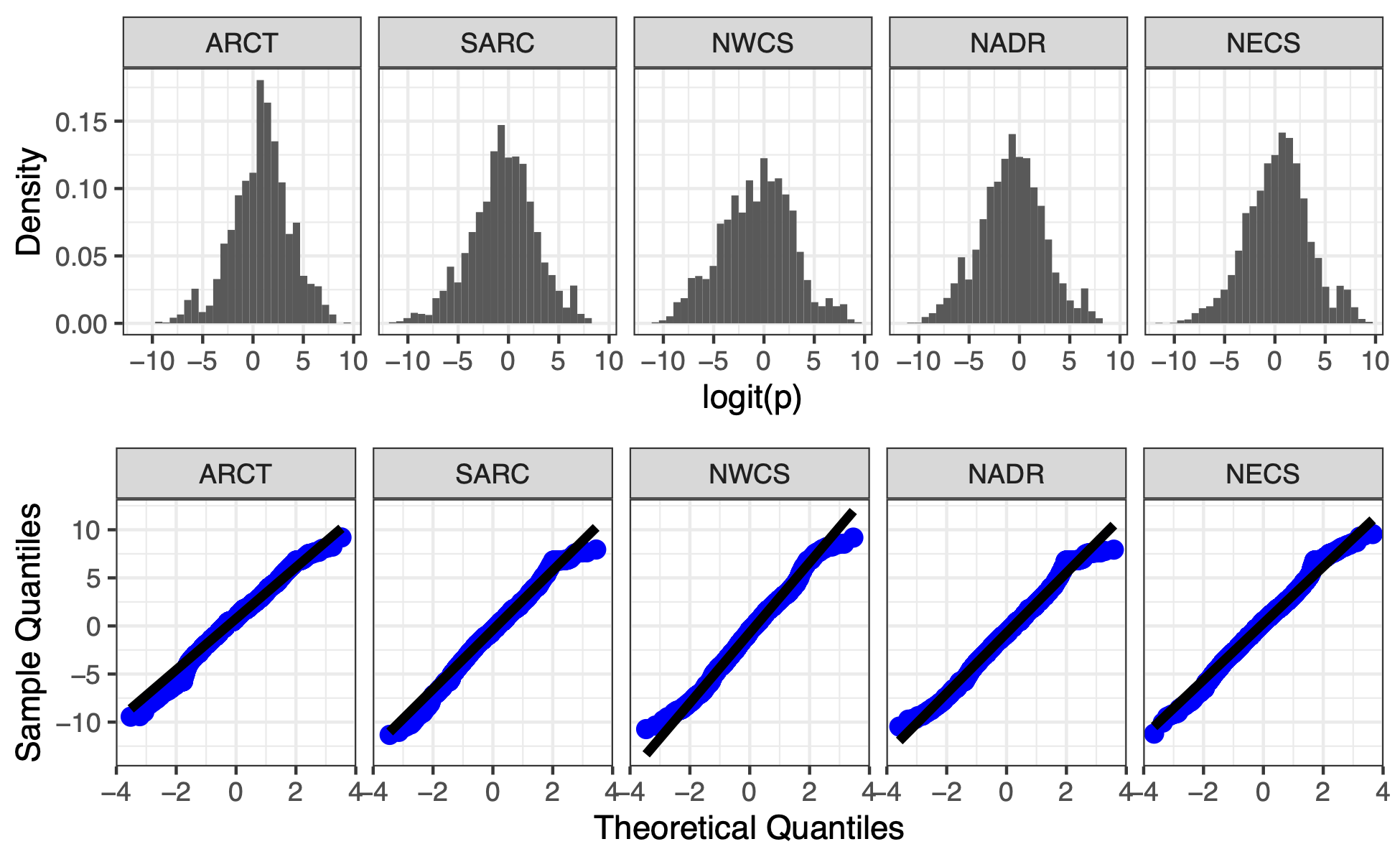

Supplement: S2 Fig — Histograms (top row) and quantile-quantile plots (bottom row) of the logit of the diatom index (diatom biomass/ (diatom + dinoflagellate biomass)) for each province showing that its distribution is approximately Normal. There are spikes at 0 and 1 in the untransformed ratios due to imputation of missing values, but these are not noticeable after the logit transform. (PNG) [file pone.0323675.s010.png]

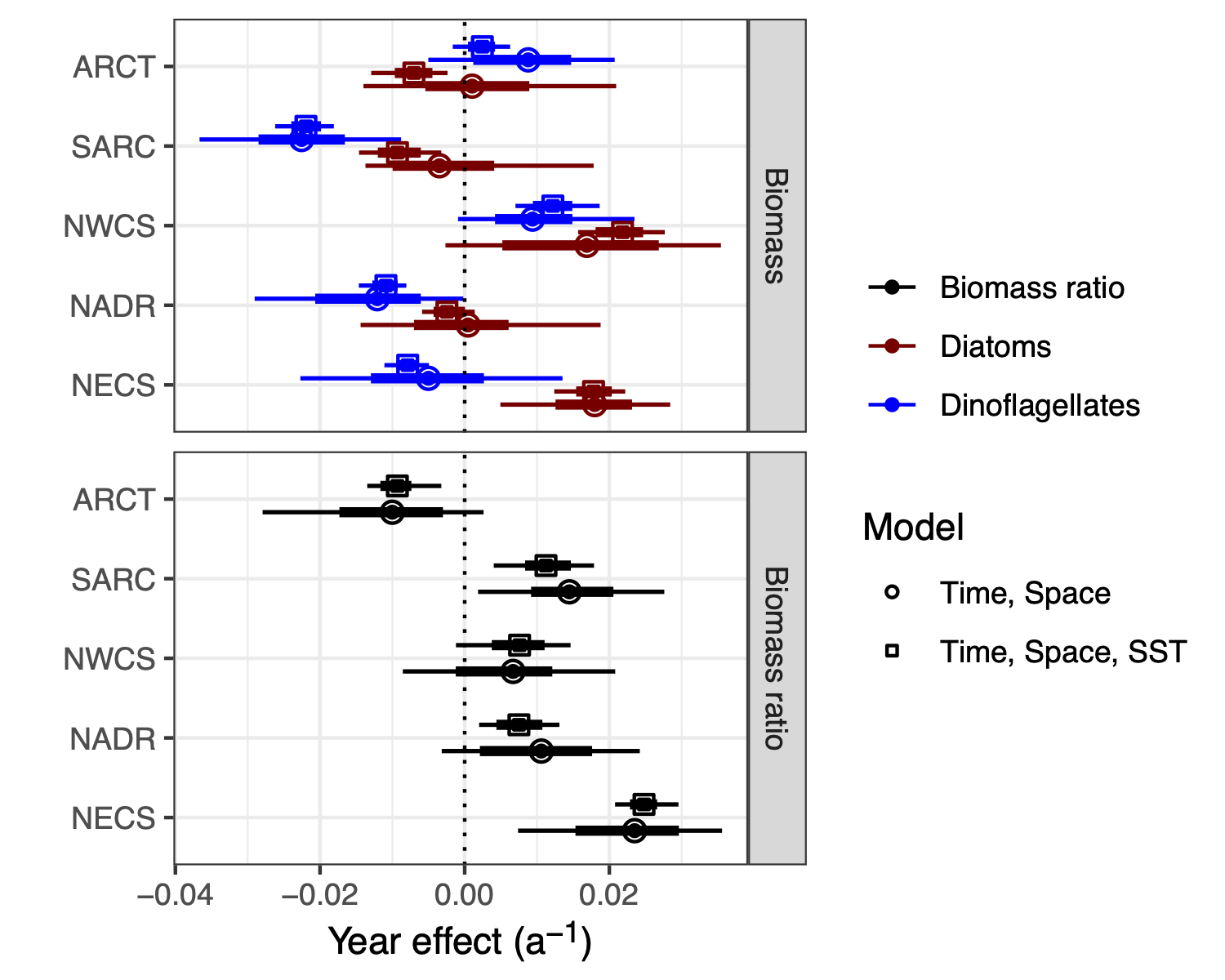

Supplement: S3 Fig — Average annual rate of change in biomass (top panel: diatoms, red; dinoflagellates blue, log scale) and the diatom index (bottom panel: diatom biomass/ (diatom + dinoflagellate biomass); logit scale) in the five biogeographic provinces estimated from two models (time, space and temperature model, squares, upper points, also shown in Fig 2; time and space, circles, lower points). An effect (slope) of 0.01 a–1 corresponds to approximately a 1% change in biomass or the biomass ratio per year. Symbols are the median of the posterior distribution and error bars are 95% (thin) and 66% (thick) credible intervals. See Fig 3 for a simplified plot with one model. (PNG) [file pone.0323675.s011.png]

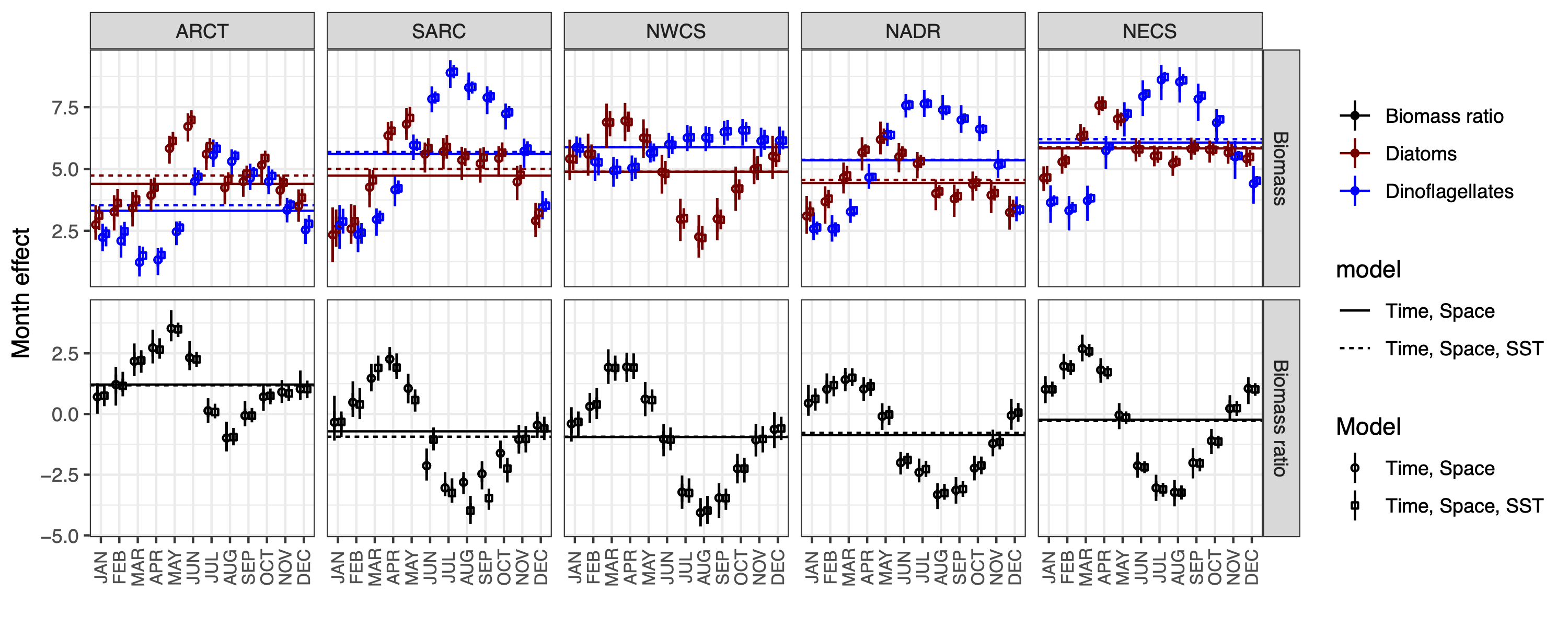

Supplement: S4 Fig — Mean monthly biomass (top panels: diatoms, red; dinoflagellates, blue; log scale) and diatom index (bottom panels: diatom biomass/ (diatom + dinoflagellate biomass); logit scale) for five biogeographic provinces estimated from two models (Time-Space, circles, left points; time-space-SST model, squares, right points). Symbols are the median of the posterior distribution and error bars are 95% credible intervals. See Fig 2 for a simplified plot with one model. (PNG) [file pone.0323675.s012.png]

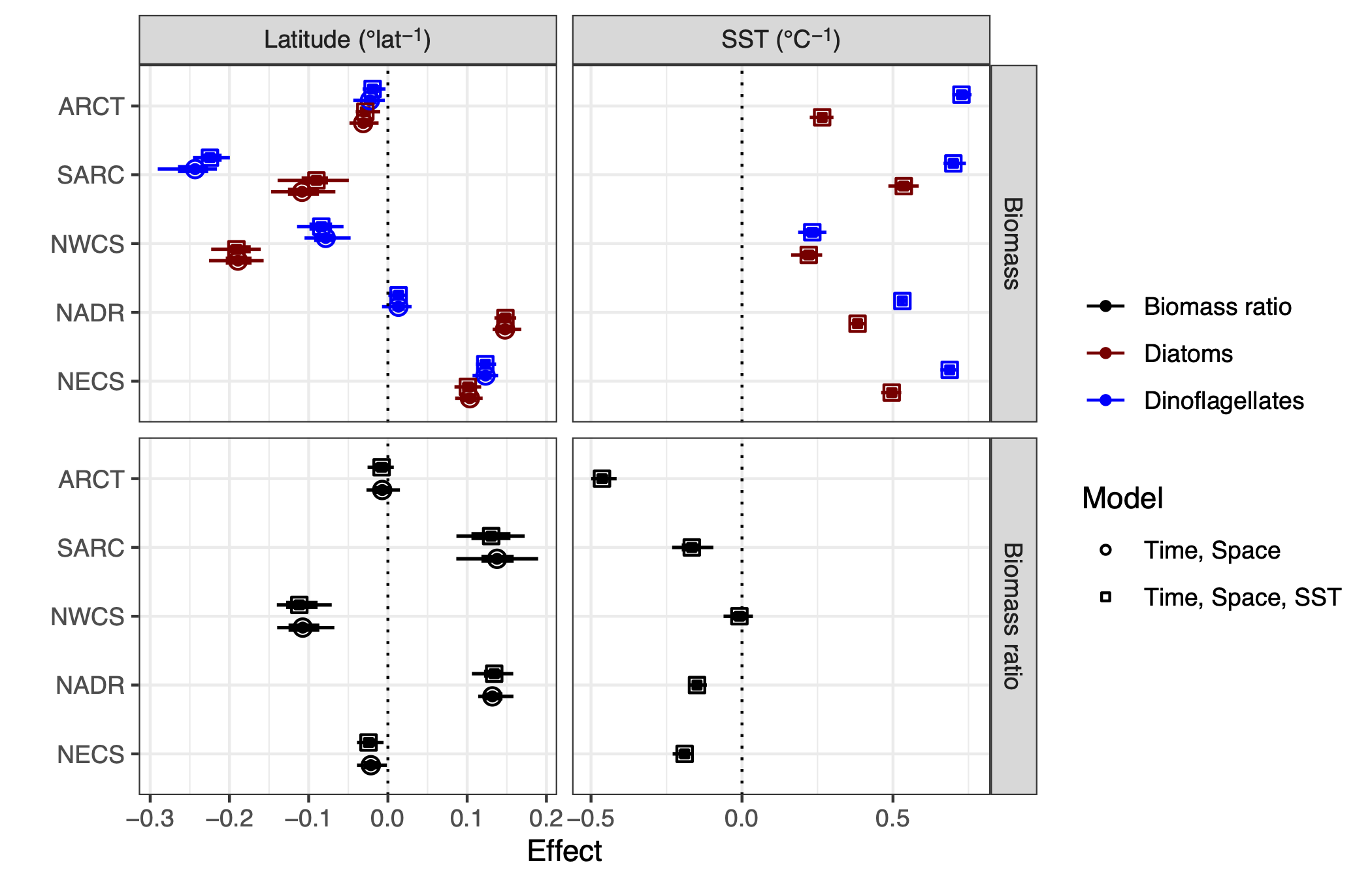

Supplement: S5 Fig — Mean effect of latitude (left panels) and temperature anomaly (right panels) for biomass (top panels: diatoms, red; dinoflagellates, blue; log scale) and biomass ratios (bottom panels: diatom biomass/ (diatom + dinoflagellate biomass); logit scale) for five biogeographic provinces estimated from two models (time and space, circles, left points; time, space and temperature model, squares, right points). An effect (slope) of 0.1 corresponds to approximately a 10% change in biomass or the biomass ratio per °latitude or °C. Vertical dashed lines emphasize 0 change. Symbols are the median of the posterior distribution and error bars are 95% (thin) and 66% (thick) credible intervals. See Fig 4 for a simplified plot with one model. (PNG) [file pone.0323675.s013.png]

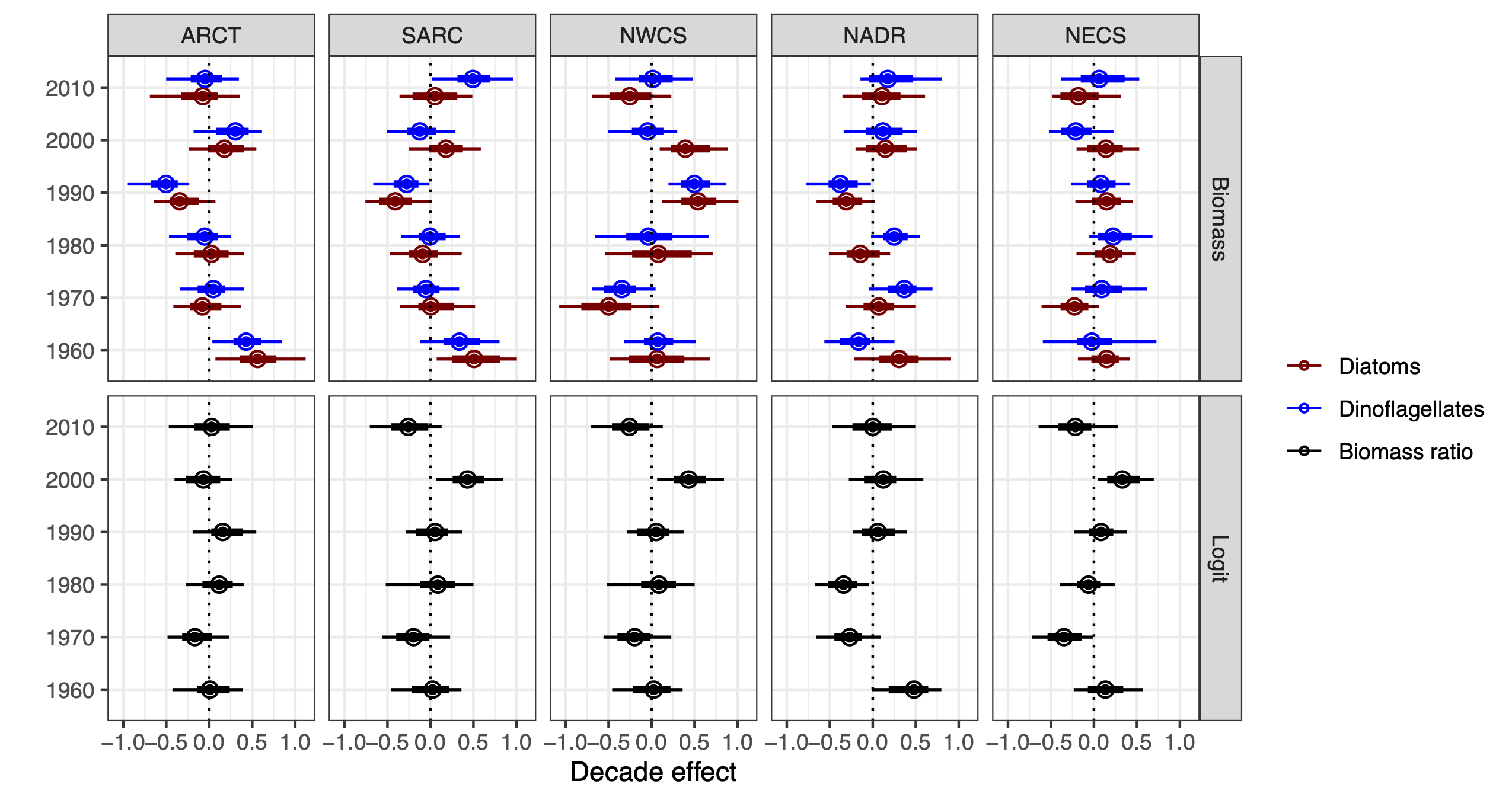

Supplement: S6 Fig — Variation in mean biomass (top panels: diatoms, red; dinoflagellates, blue; log scale) and biomass ratio (bottom panels: diatom biomass/ (diatom + dinoflagellate biomass); logit scale) for five biogeographic provinces across decades estimated from the Time-Space model. Symbols are the median of the posterior distribution and error bars are 95% (thin) and 66% (thick) credible intervals. Vertical dashed lines emphasize 0 change. (PNG) [file pone.0323675.s014.png]

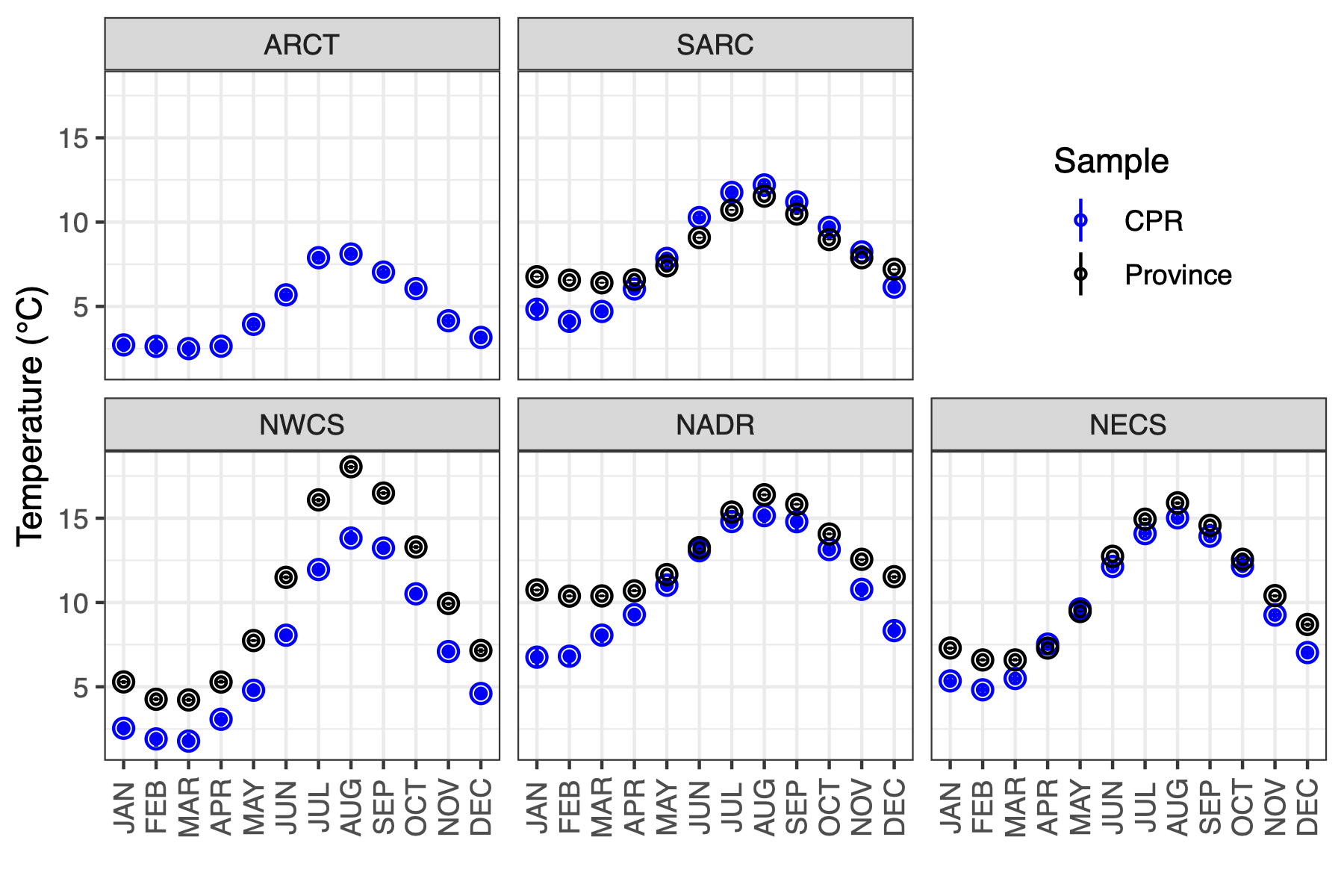

Supplement: S7 Fig — Mean monthly temperature (°C) in each biogeographic province estimated from Hadley sea surface temperature reanalysis product sampled at locations where CPR data observed (blue) and throughout each province at 1° resolution (black). Points are the median of the posterior distribution and error bars are 95% (thin) and 66% (thick) credible intervals. Error bars are all smaller than symbols. Province-wide sampling was not done for the ARCT province as its spatial extent is much larger than the CPR sampling and much of the region is at -1.8°C. (PNG) [file pone.0323675.s015.png]

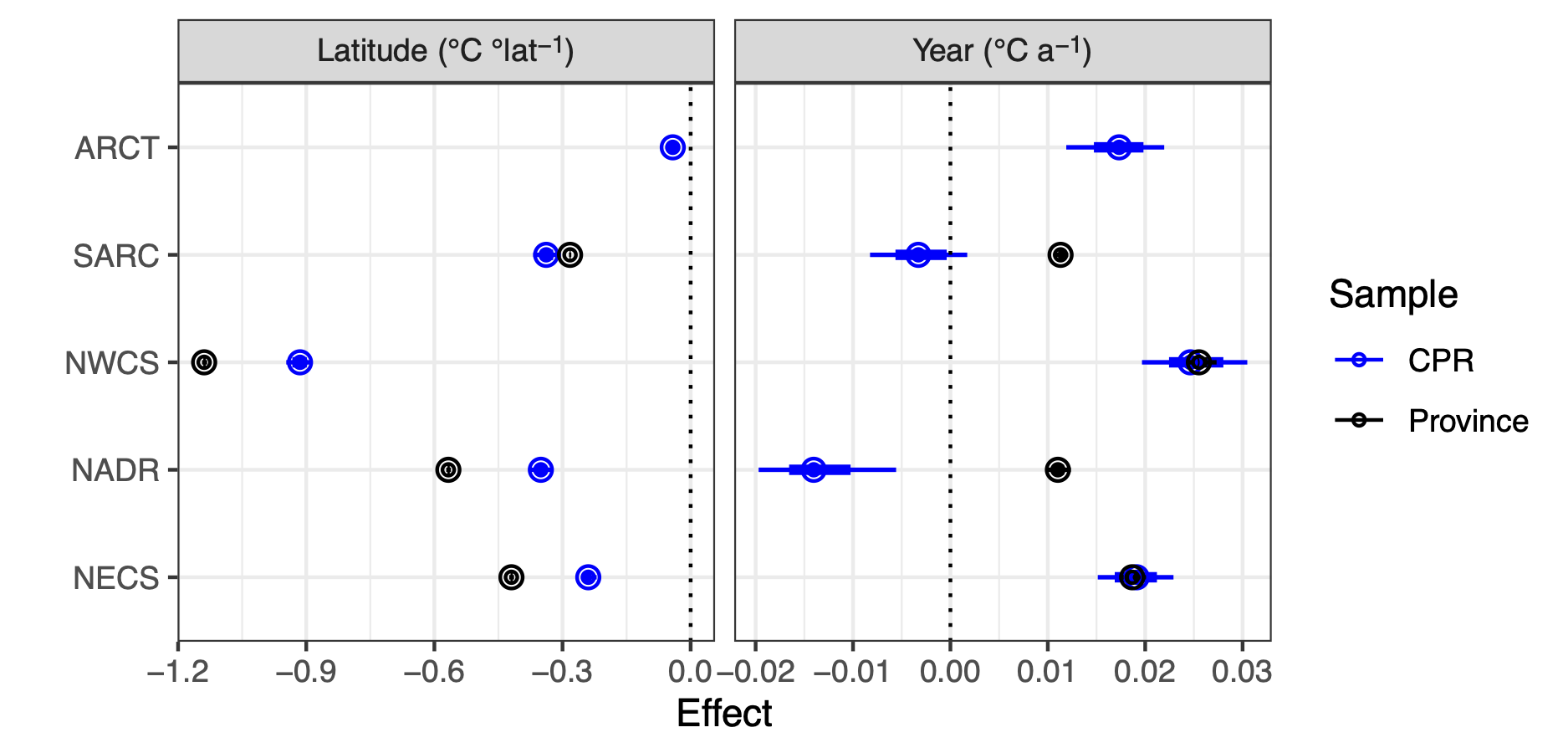

Supplement: S8 Fig — Change in mean temperature as a function of latitude (left panel, °C °lat–1) and year (right panel, °C a–1) in each biogeographic province estimated from Hadley sea surface temperature reanalysis product sampled at locations where CPR data observed (blue) and throughout each province at 1° resolution (black). Symbols are the median of the posterior distribution and error bars are 95% (thin) and 66% (thick) credible intervals. Error bars are all smaller than symbols for the latitude estimate. Vertical dashed lines emphasize 0 change. Province-wide sampling was not done for the ARCT province as its spatial extent is much larger than the CPR sampling and much of the region is at -1.8°C. (PNG) [file pone.0323675.s016.png]

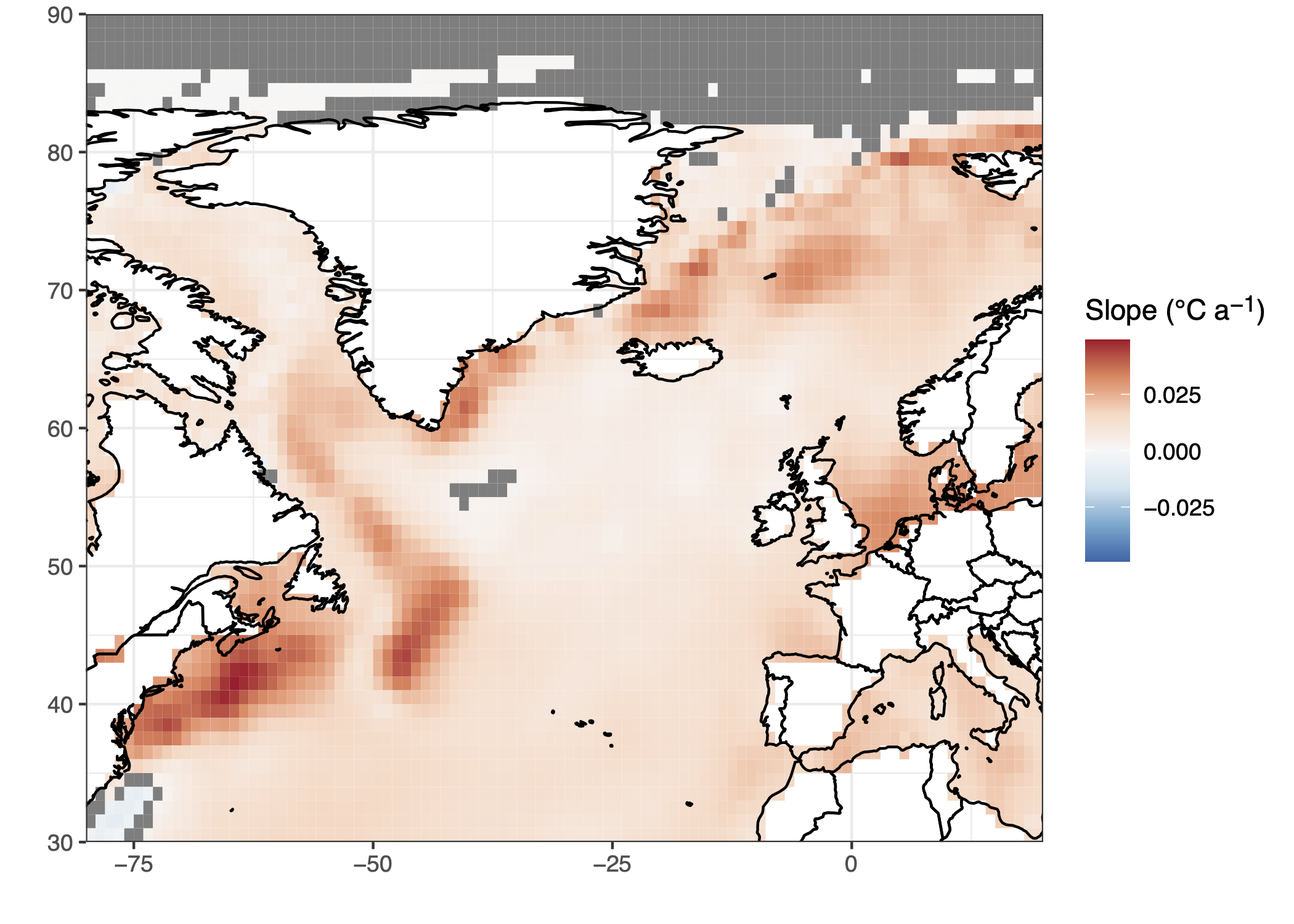

Supplement: S9 Fig — Mean rate of sea surface temperature change (°C a–1) from monthly Hadley SST over the period 1960–2017 on a 1° grid. Slopes were estimated by linear regression. Non-significant slopes (p > 0.05) masked with gray boxes. (PNG) [file pone.0323675.s017.png]

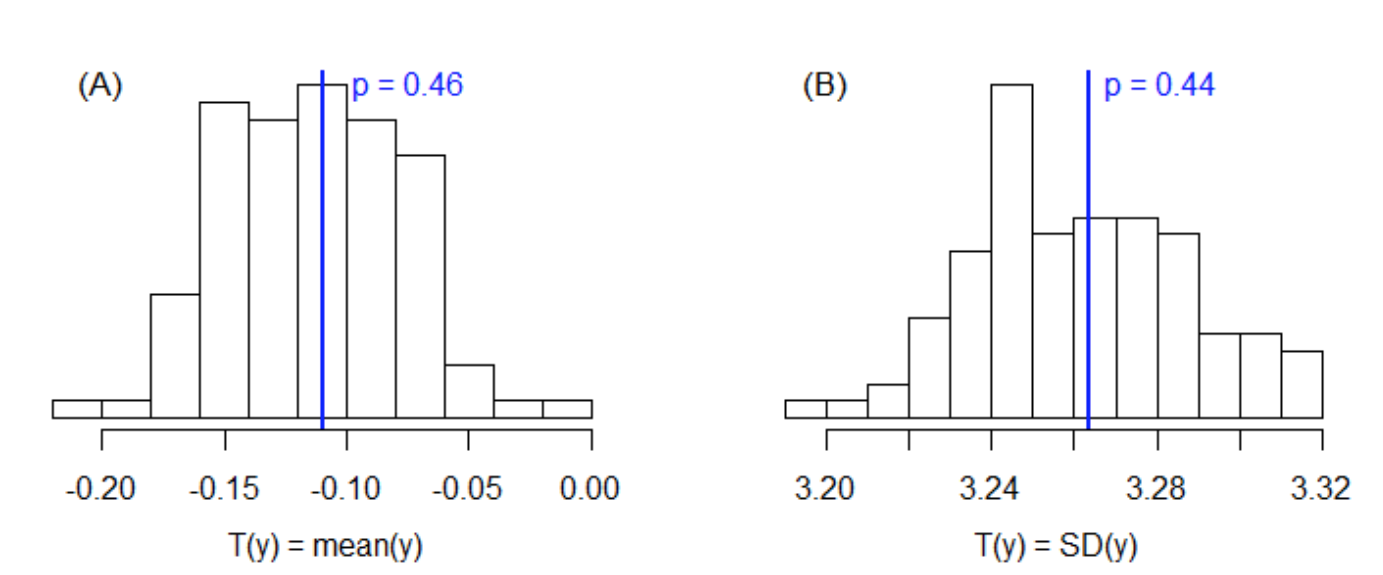

Supplement: S10 Fig — Histograms of posterior predictive means (left) and variances (right) over 100 posterior predictive of replicates for the logit of the proportion of total (diatom + dinoflagellate) biomass due to diatoms under the Time-Space-SST logit model. The vertical line represents the observed value of the test statistic. The Bayesian p-values displayed in each panel are all in the range 0.25–0.75, far from the extremes 0 and 1, implying that the model predictions do not systematically deviate from the data. (PNG) [file pone.0323675.s018.png]

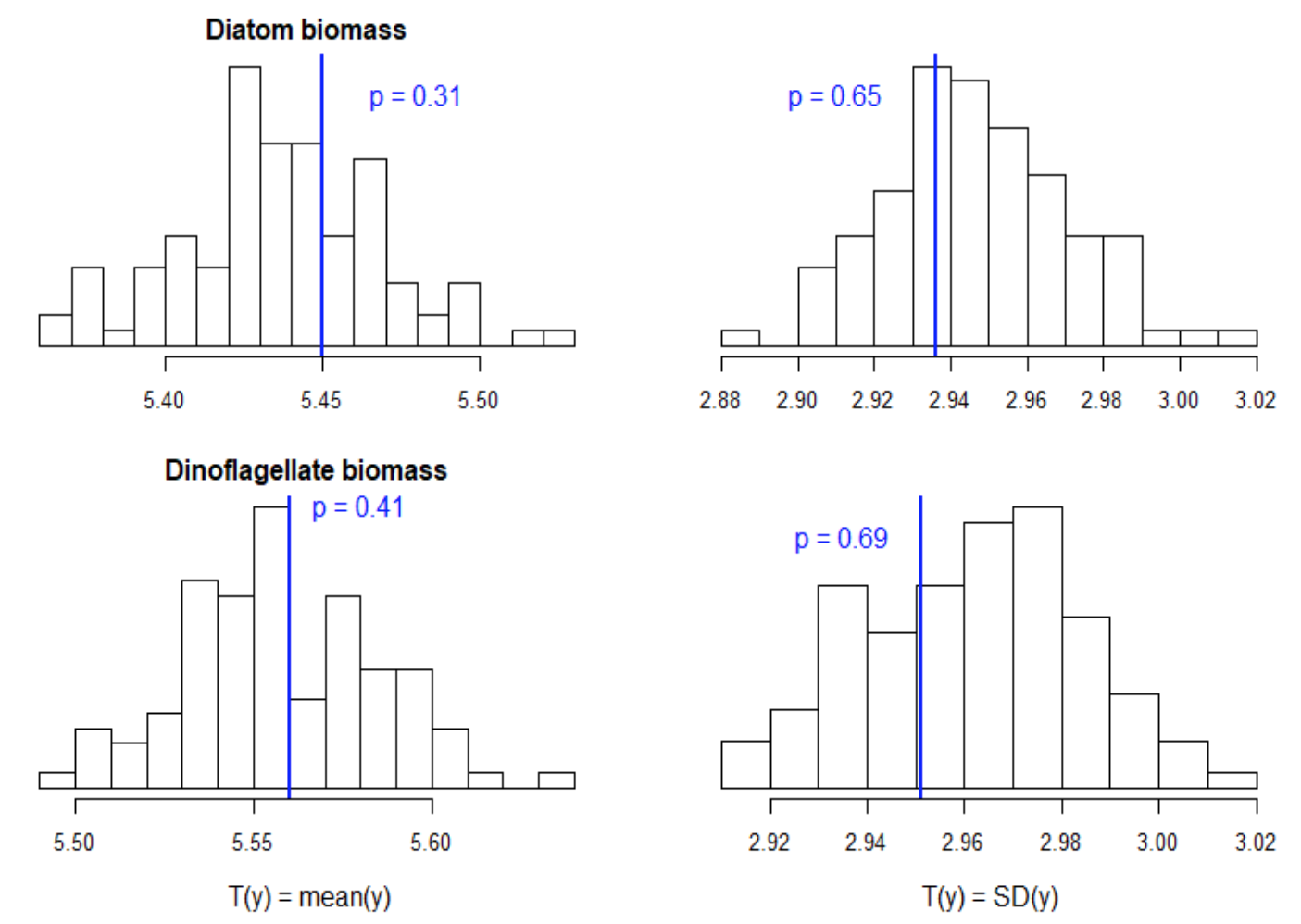

Supplement: S11 Fig — Histograms of posterior predictive means (left) and variances (right) over 100 posterior predictive data replicates for diatom (top) and dinoflagellate (bottom) log-biomasses under the Time-Space-SST biomass model. The vertical line represents the observed value of the test statistic The Bayesian p-values displayed in each panel are all in the range 0.25–0.75, far from the extremes 0 and 1, implying that the model predictions do not systematically deviate from the data. (PNG) [file pone.0323675.s019.png]
